# Ectoparasitic community of the Mahali mole-rat, *Cryptomys hottentotus mahali*: potential host for vectors of medical importance in South Africa

**DOI:** 10.1186/s13071-020-04537-w

**Published:** 2021-01-06

**Authors:** Dina M. Fagir, Nigel C. Bennett, Eddie A. Ueckermann, Alexandra Howard, Daniel W. Hart

**Affiliations:** 1 Department of Zoology and Entomology, University of Pretoria, Private Bag X20, Hatfield, 0028 South Africa; 2 Unit for Environmental Sciences and Management, Potchefstroom Campus, North-West University, Private Bag X6001, Potchefstroom, 2520 South Africa

**Keywords:** *Cryptomys*, Mole-rats, Ectoparasites, Seasonality, Fleas, *Xenopsylla*, Androlaelapid mites, Zoonotic diseases

## Abstract

**Background:**

The endemic rodent family of Bathyergidae in Africa, particularly South Africa, are understudied as reservoirs of diseases of significant medical importance. Considering the diversity and wide distribution of African mole-rats in South Africa, many of these bathyergids could act as carriers of zoonoses.

**Methods:**

The present study assessed the ectoparasite community of the Mahali mole-rat (*Cryptomys hottentotus mahali*). We aimed to identify possible parasitic arthropods that may infest this mole-rat species and explore host preference, contributions of seasonality, host sex and body mass as well as social class and colony size on ectoparasite assemblage prevalence and abundance.

**Results:**

A limited number of ectoparasite species were found on *C. h. mahali* belonging to two significant taxa: mites (Acari) and fleas, with mites being the most prevalent and abundant. We recorded the presence of *X. philoxera*, a flea well known as the principal reservoir of plague in the southern African region on the Mahali mole-rats. Only three mite species were collected: *Androlaelaps scapularis*, *Androlaelaps capensis* and *Laelaps liberiensis*. Seasonal peaks in prevalence and abundance of *X. philoxera* and *A. scapularis* were observed during summer. *Xenopsylla philoxera* abundance and *A. scapularis* loads significantly increased on reproductive mole-rat individuals in comparison to non-reproductive individuals.

**Conclusion:**

Despite the wide distribution of the subterranean African mole-rats, studies investigating their parasitic fauna remain limited and scarce. This dearth in knowledge raises the concern regarding their potential role as an endemic reservoir for zoonotic diseases. Consequently, additional sampling of their ectoparasitic community throughout their distributional range and research addressing their role as a reservoir for zoonotic diseases in southern Africa are urgently needed.
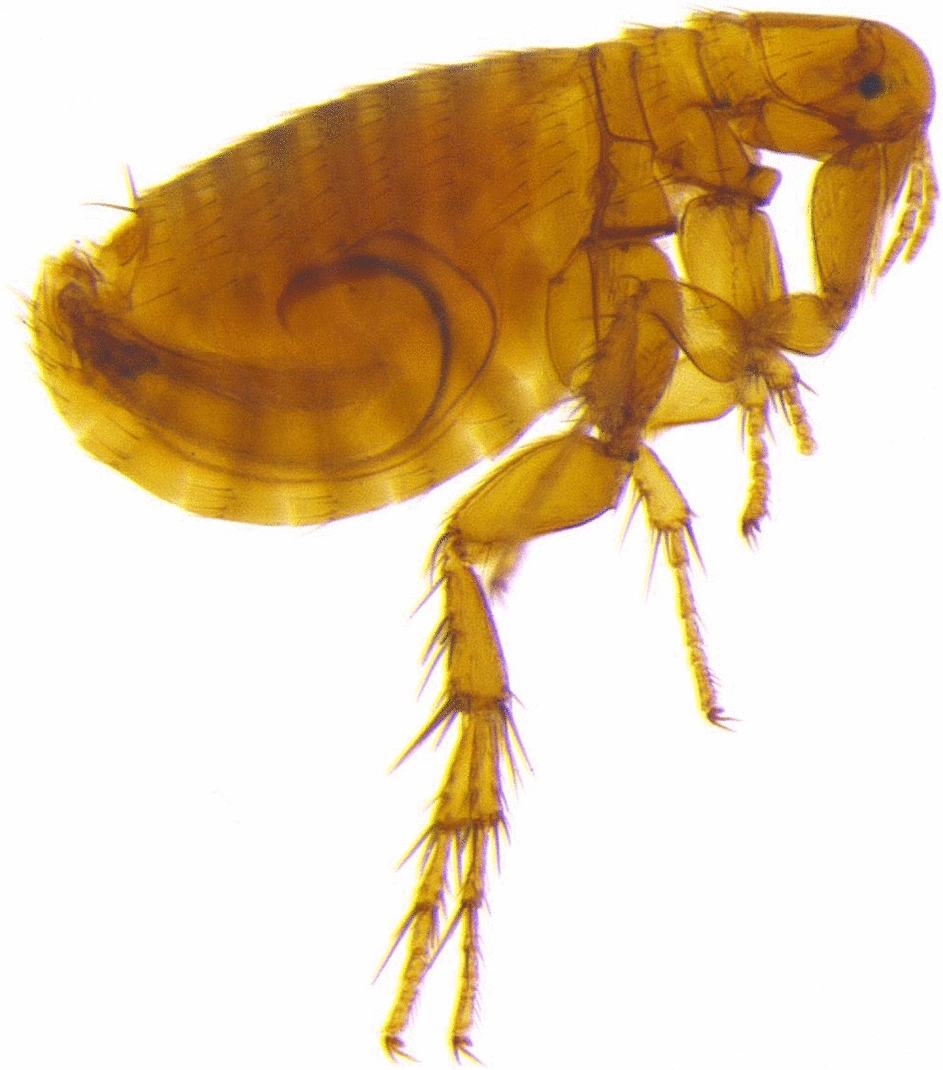

## Background

Members of the Order Rodentia globally harbour different ectoparasitic arthropods, of which many are vectors for diseases of medical and veterinary importance [1]. Factors driving parasite prevalence and abundance patterns may be divided into two general categories, environmental factors (abiotic) and host-related factors (biotic) [2, 3]. The differences in parasite burdens may result from a number of these factors (i.e. abiotic and biotic) that are not always mutually exclusive, and their contributions may be difficult to disentangle [2, 4]. Seasonal patterns can be linked to the parasite life-cycle and how, or to what degree, changes in temperature, rainfall and humidity may affect and/or determine the duration of the developmental stages (i.e. eggs, larva/nymph, adult) in these parasites, as well as the periods, spent off-host by a particular ectoparasite [5–7]. Parasite burdens often show a male-biased pattern with males being infested by more ectoparasites than females [8, 9]. Males tend to be the larger of the sexes in the majority of mammal species and to be able to sustain higher parasite numbers as they represent more substantial resources [8]. The immunosuppressive properties of testosterone, associated with breeding, could also be a contributing factor to higher male infestation [9–12]. Another host-related factor affecting parasite burdens is linked to the degree of sociality [13]. Many studies have reported an increase in parasite burdens with the increases in group size [14, 15]. In contrast, other studies failed to support this and did not find a correlation between parasite burdens and group size [16]. A study by Bordes et al. [17] suggested that a decrease in ectoparasite loads might be affected by different factors such as the host’s environment (e.g. temperature, rainfall, number of potential hosts and the number of other ectoparasites) as well as increased host defence strategies against ectoparasite transmission in social species [17–19]. Further research is required to disentangle the abiotic and biotic factors affecting differences in parasite burdens; this is particularly true in social mammalian species.

African mole-rats (family Bathyergidae) are subterranean hystricomorph rodents with a wide distributional range throughout sub-Saharan Africa [20]. Many African mole-rat species are deemed pests since they eat the roots of crops and cause property damage due to their tunnel systems. The Mahali mole-rat, *Cryptomys hottentotus mahali*, lives in colonies with only one reproductive female (queen) and up to three potential reproductive males [20]. The remaining members of the colony are reproductively inactive, with decreased levels of reproductive hormones such as testosterone and perform alloparental care to the queen’s offspring [21, 22]. The members in the colony exhibit allogrooming, which could reduce ectoparasite loads, and the movement of animals is restricted mainly by rainfall [20, 23]. A large number of studies have focused on the social system and reproductive physiology of mole-rats [21, 22, 24–26]. However, in the southern African region, particularly South Africa, very few studies have been carried out on parasites of bathyergids with only a limited number of hosts [2, 27–31]. Thus, we aimed to address this dearth of knowledge and supply an inventory list of the ectoparasites of the aseasonal breeding Mahali mole-rat. Also, the present study aimed to assess the contributions of host sex, social class, group size and season on the ectoparasitic community in the cooperatively breeding Mahali mole-rat.

## Methods

The Mahali mole-rat (*Cryptomys hottentotus mahali*) was captured on smallholdings and farms in and around the area of Patryshoek, Pretoria (25° 40′ S, 28° 2′ E), South Africa. The region of Patryshoek comprises bushveld of South Africa, characterised by cold, dry winters and hot moist summers (South African Weather Bureau). Mahali mole-rats were caught every month between October 2016 and September 2017. A total of 31 colonies were captured using Hickman live-traps baited with sweet potatoes. Traps were placed at the entrance of excavated burrows each month during the capture period and were checked every 2 h from dusk till dawn. Colonies were caught in their entirety, and a colony was deemed to be entirely trapped if no trap activity was observed for 5 consecutive days. Once captured, colonies were transported back to the mole-rat laboratory at the University of Pretoria, Department of Zoology and Entomology. Captured animals were housed with their colony mates in plastic crates (49.5 × 28 cm) with wood shavings and paper towelling provided as nesting material. The mole-rats were fed fresh sweet potato and apples daily until assessed for ectoparasites. All water requirements of the animals were satisfied in the provisioning of food.

In the laboratory, animals were weighed and sexed, and their reproductive status was determined. Reproductive males (RMs) were distinguishable from non-reproductive males (NRMs) by their large descended testes and yellow staining around the mouth. Furthermore, the RM was the heaviest male within each colony, while NRMs were significantly lighter [20]. Reproductive females (queens, RFs) were characterised by having prominent axillary teats and perforated vaginas, which were not observed in the non-reproductive females (NRFs) [32].

Individual animals were examined for ectoparasites within 5 days of capture, and a modified wash technique was applied for the assessment of ectoparasite loads [32]. Mole-rats were anaesthetised (to be used in other studies) with an overdose of isoflurane; subsequently, each individual was washed thoroughly in 100 ml of warm soapy water in a 22 × 7 × 5-cm tub to remove any ectoparasites. The washing process was standardised for all individuals and involved kneading through the fur with the fingertips all the way along and around the body length three times before washing the head and the posterior end. Subsequently, individuals were moved back and forth through the water (20 times each side), to remove any ectoparasites that remained caught in the fur. Any additional ectoparasites found on the individual after the process were also collected using tweezers. The wash water was subsequently transferred to a labelled screw-top container until processing. Each wash sample was filtered through a no. 25 US Standard Sieve (710-|j.micron screen) for ectoparasites, and these were then collected and placed into Eppendorf tubes containing 70% ethanol for preservation. For microscopic examination, mites were cleared and mounted following the techniques described in [33]. Fleas and mites were identified using the morphological keys of [33–35].

Prevalence and mean abundances were calculated for the two higher taxa (i.e. mites and fleas) and the most common individual parasite species. The rarity of some parasite species precluded any meaningful statistical analyses; therefore, only descriptive statistics were reported for the rare parasite species. Data were not normally distributed even after log-transformation (Kolmogorov–Smirnov test: *P* < 0.0001). The effect of the four seasons, i.e. spring (September, October, November 2016, September 2017), summer (December 2016, January, February 2017), autumn (March, April, May 2017), and winter (June, July, August 2017), host sex, host body mass, individual social class (i.e. reproductive and non-reproductive animals) as well as colony size on variation in prevalence and abundance of the ectoparasites were investigated using generalized linear models (GZLMs). A model with a binomial distribution with a logit-link function was selected for prevalence data and a negative-binomial distribution with a log-link function for abundance data. *Post hoc* analyses were performed with a pairwise comparison using the least significant difference (LSD). All two-way interactions were included in the model, and since none of them yielded a significant result, only main effects were reported in the present study. For meaningful statistical analyses, all different developmental stages of parasite species (i.e. larva, nymph, male, female) were pooled for analysis. We tested the effect of colony size, average temperature and rainfall on ectoparasite burdens in a separate GZLM to avoid parameters overload on the model. All statistical analyses were conducted in IBM SPSS version 25 (IBM SPSS Statistics 25. Ink 2017), and results are reported as the mean ± standard error (SE).

## Results

A total of 202 individual mole-rats were captured and assessed for ectoparasite loads (Table 1). The overall prevalence of ectoparasites was 69.3% (140/202 individuals), with a total of 1395 individual ectoparasites (mean abundance 6.91 ± 1.26) collected from these mole-rats. The ectoparasites collected belonged to two major taxa, fleas and mites, with the androlaelapid mites being the most prevalent and abundant ectoparasites (Table 2). Neither ticks nor lice were found on any mole-rat individuals.

**Table 1:** Summary of individual Mahali mole-rats (*Cryptomys hottentotus mahali, n* = 202) captured per season

Season	No. of NRF (%)	No. of RF (%)	No. of NRM (%)	No. of RM (%)	Total (%)
Spring 2016	20 (9.9)	3 (1.5)	12 (5.9)	4 (2.0)	39 (19.3)
Summer 2016/2017	29 (14.4)	6 (3.0)	19 (9.4)	9 (4.5)	63 (31.2)
Autumn 2017	10 (5.0)	4 (2.0)	9 (4.5)	5 (2.5)	28 (13.9)
Winter 2017	21 (10.4)	4 (2.0)	28 (13.9)	7 (3.5)	60 (29.7)
Spring 2017	4 (2.0)	1 (0.5)	4 (2.0)	3 (1.5)	12 (5.9)
Total	84 (41.6)	18 (8.9)	72 (35.6)	28 (13.9)	202 (100)

*NRF* non-reproductive female, *RF* reproductive female, *NRM* non-reproductive male, *RM* reproductive male

**Table 2:** Ectoparasites observed on Mahali mole-rats (*Cryptomys hottentotus mahali*) in Patryshoek area north of the Magaliesburg mountain, South Africa, over 1 calendar year

Species	L	PN	DN	M	F	Total	Prevalence (%)	Mean abundance ± SD
Fleas								
* Xenopsylla philoxera*	–	–	–	47	37	84	23.3	0.42 ± 1.000
Mites								
* Androlaelaps scapularis*	1	125	241	113	806	1286	62.4	6.37 ± 17.751
* Androlaelaps capensis*	0	0	3	1	18	22	8.4	0.11 ± 0.397
* Laelaps liberiensis*	0	1	0	2	0	3	1.0	0.01 ± 0.157

*L* larva, *PN* protonymph, *DN* deutonymph, *M* male, *F* female, *SD* standard deviation

**Fig. 1:**
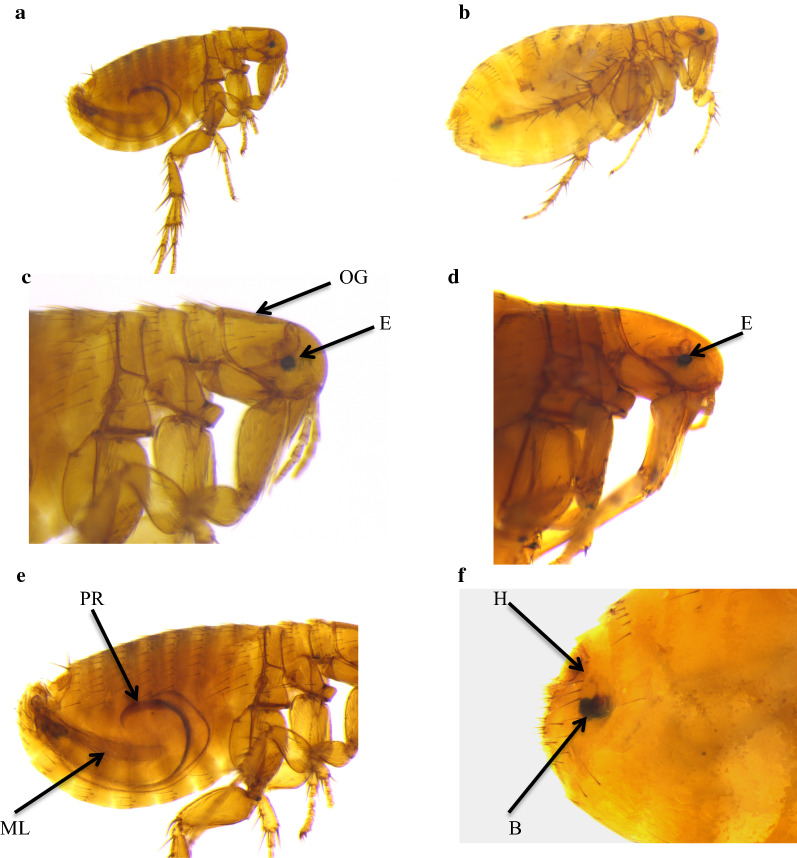
*Xenopsylla philoxera* (flea). **a** Male, whole body. **b** Female, whole body. **c** Male, head (note the well-developed and pigmented eye (E) and the shallow occipital groove (OG). **d** Female, head (note the well-developed and pigmented eye (E)). **e** Male, abdomen (phallosome: median lamina (ML) and penis rod (PR). **f** Female, abdomen (spermatheca: bulga (B) and hilla (H))

**Fig. 2:**
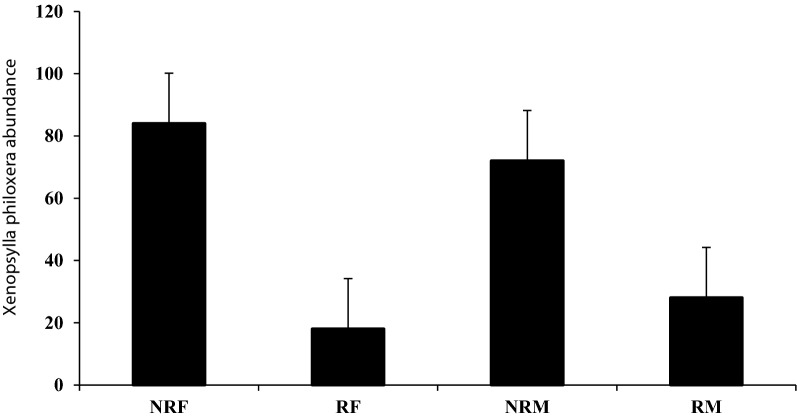
Effect of host reproductive status on the abundance of *Xenopsylla philoxera*

All fleas were identified as *Xenopsylla philoxera* (Fig. 1). Flea prevalence differed significantly with season, and it was significantly lower in winter (14.9%, *n* = 7) compared to spring (29.8%, *n* = 14, LSD: *P* ≤ 0.043) and summer (44.7%, *n* = 21, LSD: *P* = 0.003). Host sex, host body mass, individual status and colony size had no significant effects on flea prevalence (Table 3). Flea abundance was significantly higher in summer (mean 0.63 ± 0.128) compared to autumn (mean 0.29 ± 0.115, LSD: *P* = 0.042) and winter (mean 0.17 ± 0.058, *P* = 0.001). In addition, flea abundance was higher in spring (mean 0.50 ± 0.120) than in winter (mean 0.17 ± 0.058, *P* = 0.013). Reproductive status of the mole-rat significantly affected the flea abundance, with reproductive males and females harbouring more fleas than non-reproductive ones (Fig. 2). In contrast, sex, body mass and colony size had no significant effect on the abundance of fleas (Table 3).

**Table 3:** Results of the GLMs for total mite and flea prevalence and abundance of the Mahali mole-rats (*Cryptomys hottentotus mahali*) over one calendar year

Parasite	Factor	Prevalence			Abundance		
		Wald *χ*^2^	*df*	*P*	Wald *χ*^2^	*df*	*P*
*Xenopsylla philoxera*	Season	8.215	3	0.042*	12.513	3	0.006*
	Sex	1.178	1	0.278	0.036	1	0.849
	Body mass	0.000	186	1.000	10.719	45	1.000
	Animal status	3.218	1	0.073	9.242	1	0.002*
	Colony size	13.592	12	0.328	16.732	12	0.160
*Androlaelaps scapularis*	Season	15.782	3	0.001*	146.379	3	< 0.0001*
	Sex	0.011	1	0.916	0.181	1	0.670
	Body mass	0.000	186	1.000	166.343	124	0.007*
	Animal status	5.364	1	0.021*	1.862	1	0.172
	Colony size	0.000	1	1.000	2.660	1	0.103

^*^Significant results

**Fig. 3:**
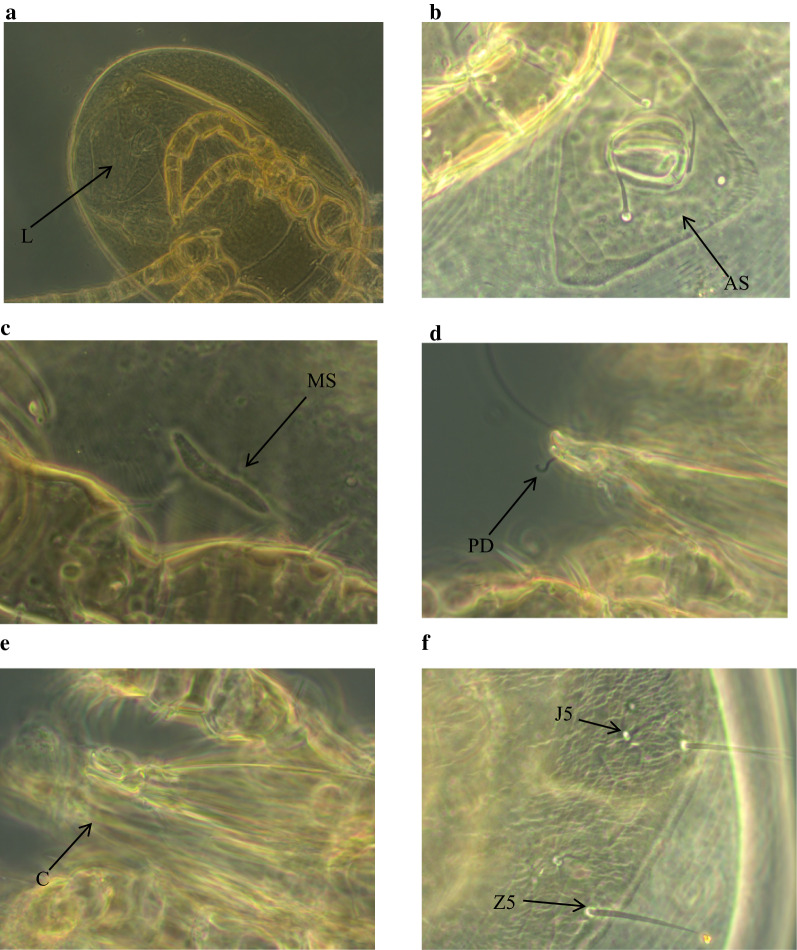
Female *Androlaelaps scapularis* (mite). **a** Opisthogaster with larva (L) inside body (arrow). **b** Anal shield (AS). **c** Metapodal shield (MS). **d** Pilus dentilis (PD). **e** Chelicera (C). **f** Dorsal shield showing seta Z5 much longer than seta J5

**Fig. 4:**
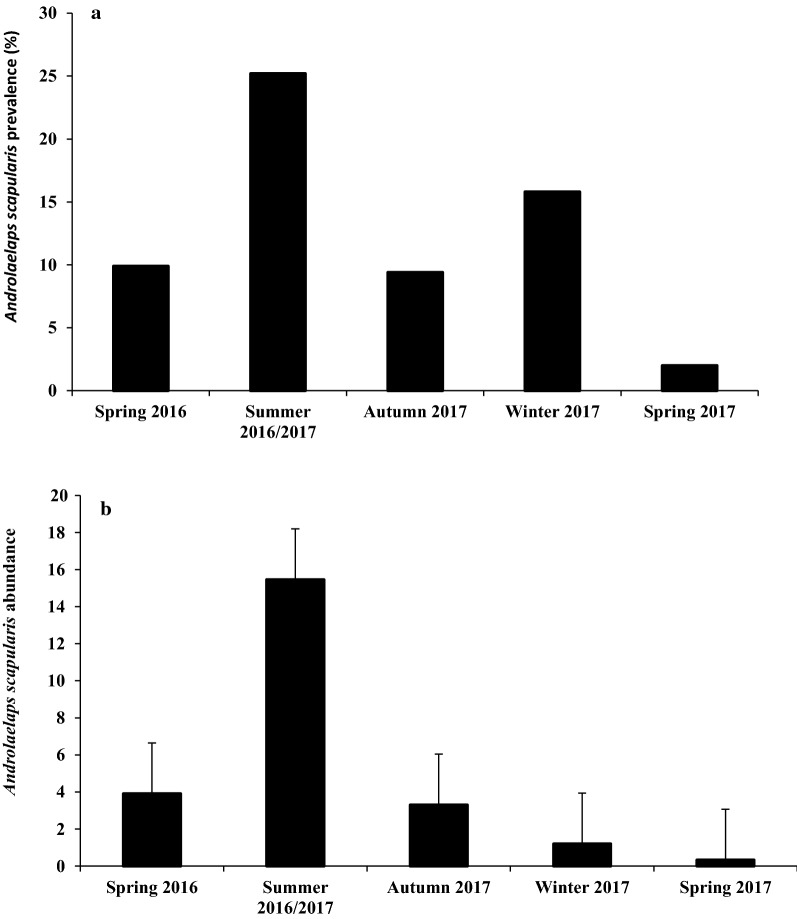
Seasonal variations in *Androlaelaps scapularis* on *Cryptomys hottentotus mahali* in over one calendar year. **a** Prevalence. **b** Abundance

**Fig. 5:**
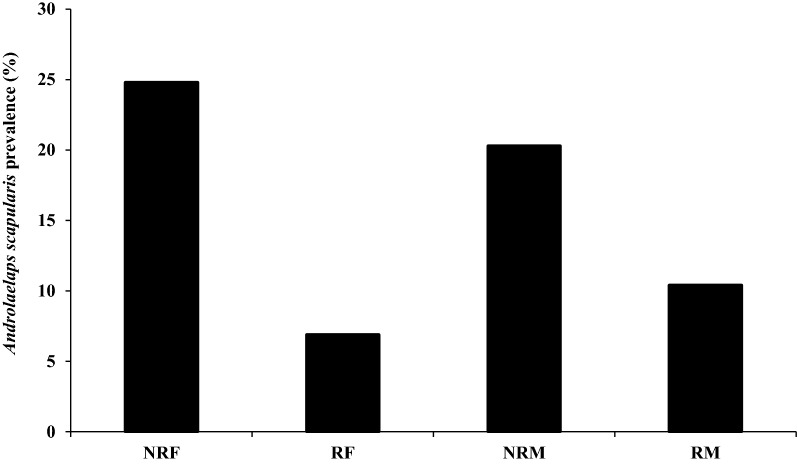
Effect of host reproductive status on the prevalence of *Androlaelaps scapularis*

The mesostigmatic mite, *Androlaelaps scapularis*, was the most prevalent and abundant species (Table 1, Fig. 3). The majority of *A. scapularis* collected were adults with a bias towards females. Among all individuals captured, *A. scapularis* prevalence was 62.4% and did not vary significantly with host sex, nor with the body mass or colony size (Tables 2,3). However, *A. scapularis* prevalence varied significantly with the seasons and animal status (Tables 2, 3). *Androlaelaps scapularis* prevalence was significantly higher in summer compared to spring, autumn and winter (Fig. 4a, Table 3). Animal reproductive status had a significant effect on *A. scapularis* prevalence (Table 3). There was no significant difference between the sexes reproductive status, but the non-reproductive individuals (males and females) were more infested by mites compared to reproductive individuals (Fig. 5, Table 3). Mean abundance of *A. scapularis* was 6.37 ± 1.249, and it varied significantly with season (Table 3). *Post hoc* analyses showed that mite abundance was significantly higher in summer compared to spring, autumn and winter (Fig. 4b). Host body mass had a significant effect on mite abundance; however, *post hoc* analyses did not confirm these results (LSD: *P* ≥ 0.731). In contrast, host sex, reproductive status and colony size had no significant effect on the abundance of *A. scapularis* (Table 3).

## Discussion

The parasite assemblage found in the present study was limited to two arthropod taxa (fleas and mites) with a low species diversity. Similar low parasite species diversity has been recorded for several other subterranean rodents [2, 7, 20, 36]. The reported low parasite burden may be a result of the subterranean lifestyle of the study species, which could limit exposure to parasites [31, 37–40]. *Xenopsylla philoxera* has not previously been reported from any South African mole-rats, although another flea species (*Cryptopsylla ingrami*) has been reported from *C. h. hottentotus* [37]. The two mites *A. scapularis* and *A. capensis* have been reported in previous studies from *Cryptomys hottentotus hottentotus*, *C. h. pretoriae* and *Fukomys damarensis* [31, 36, 37, 39]. On the Mahali mole-rat, as well as on closely related species (*C. h. hottentotus* and *C. h. pretoriae*), mites were the dominant taxa [31, 37].

*Androlaelaps scapularis* was observed to be the most prevalent and abundant parasite species, while the prevalence and abundance of *A. capensis* were slightly lower in this present study compared to those of *C. h. hottentotus* [37]. However, the low prevalence and abundance of parasites on Mahali mole-rats are similar to those of *C. h. pretoriae* [31]. Both of these mite species have only ever been recorded from bathyergids, indicating host specificity at the family level [31, 35]. Clear seasonal patterns were found for both prevalence and abundances of overall mites and fleas. The burdens of both mites and fleas were higher during the wet summer, as reported for the highveld mole-rat [31]. In contrast, in the common mole-rat (*C. h. hottentotus*), ectoparasite burdens were higher during the wet winter [37], indicating that changes in humidity rather than temperature are responsible for seasonal variations in ectoparasite burdens. Seasonal variations and the importance of humidity for ectoparasites are well known for various taxa [34]. Seasonal variations in parasite loads may also be a result of a synchronized reproductive cycle between parasite species and their hosts [34, 35]. The lack of sex bias in the present study may be linked to the sedentary lifestyle of the study species. The same observation has been previously observed in a closely related species (*C. h. pretoriae*) [28]. This can be linked to the shared habitat of group members of different sex and reproductive status [31]. Also, activities such as locomotion and dispersal are energetically costly underground; therefore, these activities are often restricted to rainfall seasons when burrow digging is less costly [31]. This results in members of a colony sharing the same burrow system for extended periods; thus, as a result, all colony members have similar parasite exposure regardless of their sex [31].

This study, interestingly, revealed that there were reproductive status differences in parasite loads. Reproductive individuals are likely to be older in age, and the lower parasite abundance in these individuals may be an indicator of acquired immunity against parasitic infestation [31]. No significant pattern was found between parasite load and colony size in the study population. Contrastingly, Bordes et al. [18] and Viljoen et al. [31] found a significant relationship between group size and parasite loads. Two possible reasons are put forward to explain the lack of significant differences between parasite abundance and group size in our study. First, the host environment may affect parasite burdens [19]. Host environment refers not only to the abiotic factors, such as temperature and rainfall, but also it extends to some host biotic factors, such as the number of potential hosts and number of other parasite taxa/species, which may affect competition between parasites over space or nutrition [18]. The second hypothesis suggests that social groups may have developed defensive strategies to control parasite transmission, such as increased allogrooming rates in larger groups [18, 19].

## Conclusions

In summary, the ectoparasitic fauna of the Mahali mole-rat was limited to a small number of parasite species and dominated by one mite species. The *Androlaelaps* mites appear to be specific to the Bathyergidae, whereas the flea appears to be a generalist. Both prevalence and abundance of mite and flea exhibited seasonal peaks in summer.

## Data Availability

Data and materials were made available by NCB and DWH.

## Abbreviations

NRF: Non-reproductive female
RF: Reproductive female
NRM: Non-reproductive male
RM: Reproductive male
L: Larva
PN: Protonymph
DN: Deutonymph
M: Male
F: Female
SD: Standard deviation
